# Database of fish fauna in a highly urbanised river (Tsurumi River Basin, Kanagawa, Japan)

**DOI:** 10.3897/BDJ.10.e83527

**Published:** 2022-05-18

**Authors:** Rei Itsukushima, Keisuke Maruoka

**Affiliations:** 1 Tokyo Institute of Technology, Yokohama, Japan Tokyo Institute of Technology Yokohama Japan

**Keywords:** urban stream, fish fauna, river channel modification, rare species, non-native species

## Abstract

**Background:**

Urbanisation has facilitated changes in the hydrological cycle, sediment dynamics and habitat loss and it has had a tremendous impact on river ecosystems. Moreover, the invasion of non-native species reduces the number of native species with the progress of urbanisation, thereby resulting in the homogenisation of fish fauna and significant decrease in diversity. However, the effects of urbanisation on fish fauna vary depending on the region, degree of urbanisation and number of years since the construction of the city. Hence, it is necessary to collect information on how fish fauna changes due to urbanisation in different regions. The target site of the present study is the Tsurumi River, of which approximately 85% of the basin is urbanised and many of the river channels have been affected by straightening and concrete channelling. Monitoring of biota has been conducted mainly in downstream areas; however, data on fish fauna in upstream areas and tributaries of the river, which have been substantially affected by urbanisation, are lacking.

**New information:**

Surveys were conducted at 71 sites in the Tsurumi River Basin during summers and winters, focusing on river channels that have been straightened or converted to concrete channels due to urbanisation. As a result of this investigation, 10 families, 28 species and 9,335 individuals were collected. Some of the fish collected included rare species, such as *Tanakialanceolata*, *Lefuaechigonia* and *Pseudobagrustokiensis*, indicating that, even in rivers that have been severely impacted by human activities, there are still rare indigenous species living there, albeit in limited numbers. In addition, *Misgurnus* sp. (clade B2), *Micropterussalmoides*, *Lepomismacrochirus* and *Poeciliareticulata* were confirmed to be non-native species. Misgurnus sp. (clade B2) was widespread; however, populations of carnivorous non-native alien species were small. The data are all accessible from the document “database_fish_urban_tsurumi” (https://ipt.pensoft.net/manage/resource?r=database_fish_urban_tsurumi).

## Introduction

The concentration of population from suburbs to urban areas increased rapidly in the 20th century; the global urban population was about 220 million, (14% of the total population) in 1900, whereas more than half of the total population, about 3.9 billion people, lived in urban areas in 2015 (Napieralski and Carvalhaes 2016). The concentration of the population in urban areas is expected to further increase and, by 2050, it is expected to reach 6 billion or two-thirds of the total population (UNPD (United Nations, Population Division) 2014). Infrastructure development corresponding to population growth causes major modifications in urban rivers (Eyles 1997). Channel modifications that occur in urban areas worldwide change flow velocity and nutrient (such as carbon and nitrogen) transport in the channel (Catford et al. 2007, Wenger et al. 2009, Beaulieu et al. 2014), habitat degradation (Meyer et al. 2005a, Elmore and Kaushal 2008), thereby hindering the movement and dispersion of aquatic organisms (Meyer et al. 2005b) and altering biota (Smith et al. 2009, Neale and Moffett 2016). The physical, chemical and biological degradation of urban streams has been termed the "urban stream syndrome" (Walsh et al. 2005). Habitat loss due to urbanisation has resulted in a decline in the species diversity of diverse taxa (Blair and Launer 1997, McKinney 2002, Kowarik 2011). Amongst them, aquatic organisms are affected by urbanisation, habitat loss due to river channel modification and the invasion of exotic species (Pauchard et al. 2006, Engman and Ramírez 2012). In general, it has been reported that, in areas where alien species have invaded and native species have declined, homogenisation of the fish fauna has occurred and diversity has been greatly reduced (Marchetti et al. 2006, Olden et al. 2006).

In Japan, the target area of this study, urban areas have been formed in various regions since the modern era, whereas many large cities have been formed because of population influx and rapid economic growth associated with changes in industrial structure (Fujii 1990). In the Tokyo Metropolitan Area, many river channels have been buried and the remaining channels have been affected by river channel modification and about 40% of the river channels have been straightened (Itsukushima and Ohtsuki 2021). However, there are very few studies that reflect the current status of urban fish fauna or compare it with that of suburban fish fauna. One study has reported that freshwater fishes in the Tatara River, which flows through Fukuoka City, have been declining as the ratio of urban land use increases, with a particularly marked decline in *Acheilognathus* (Onikura et al. 2006). Nonetheless, the accumulation of basic data on fish fauna of a region is essential for river revitalisation and ecosystem conservation in urban rivers. This has been gaining momentum in recent years.

The Tsurumi River, which flows through Tokyo and Kanagawa Prefectures in Japan, has been urbanised since the 1950s and 85% of the River Basin now comprises urban areas. Monitoring of fish fauna has been conducted by the government since the 1990s, mainly in the lower reaches of the main river; however, surveys have not been conducted in tributaries of the region. These tributaries have witnessed considerable environmental changes, but information on fish fauna is presently lacking. This paper reports the results of a survey of the fish fauna of a tributary river for which information is lacking and provides important basic knowledge for environmental conservation and river revitalisation of small and medium-sized urban rivers.

## Sampling methods

### Study extent

**Figure 1.**
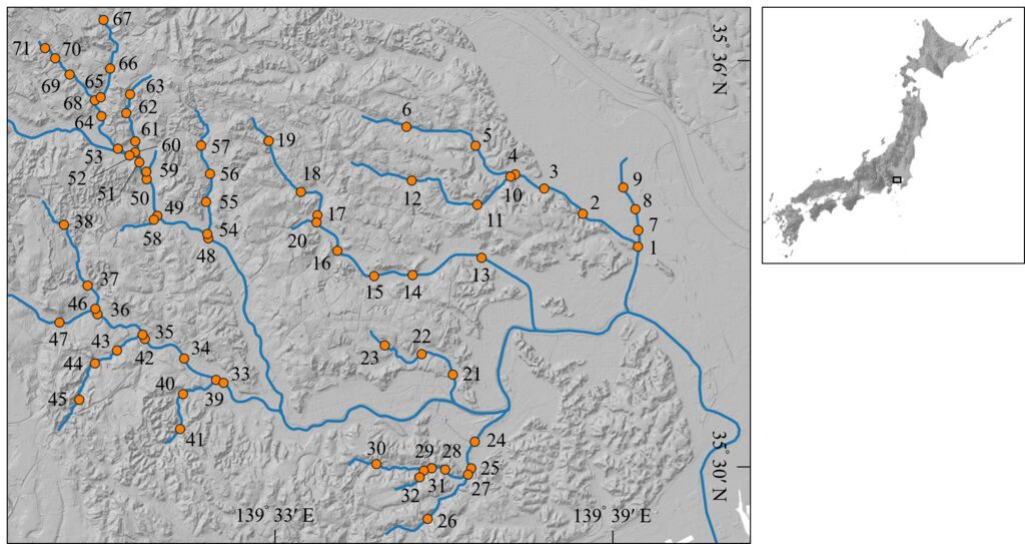
Location of the study site.

This study was conducted on the Tsurumi River, which flows through Tokyo and Kanagawa Prefectures in Japan (length of the main river channel: 43 km, Basin area: 235 km^2^). The Tsurumi River flows through a large metropolitan area and the land use in the Basin is approximately 85% urban and 15% forest and farmland, with overcrowded urban areas distributed throughout the Basin. The urban area ratio was approximately 10% in 1960; however, the urban area ratio was 85% and the population density reached approximately 8,000 people/km^2^ on average in the Basin in 2002 (Ministry of Land, Infrastructure, Transport and Tourism 2005). The investigation of fish fauna was conducted at 71 stations in the Tsurumi River Basin (Fig. 1), mainly in environmentally-degraded tributaries where data on fish fauna have not yet been collected.

### Sampling description

The fish survey was conducted using a pulsed DC Smith-Root Model LR-24 backpack electrofisher (Smith-Root Inc., Vancouver, WA, U.S.A.) at each habitat (rapid, run, pool, glide, slack and backwater) of the 71 stations. A hand net (40 cm wide, 2 mm mesh) was used to collect fishes paralysed by the electric current from the electroshocker. Surveys were conducted by two to three persons per site for a period of at least 30 minutes. The length of the survey section was one reach (approximately ten times the width of the river channel).

### Step description

In this study, we recorded occurrence data, which were identified on-site and in the laboratory according to Kawanabe and Mizuno (1989) and Seno (2007).

## Geographic coverage

### Description

Surveys were conducted at 71 sites in the Tsurumi River Basin during summers and winters, focusing on river channels that have been straightened or converted to concrete channels due to urbanisation.

### Coordinates

35.4884 and 35.6095 Latitude; 139.4807 and 139.6580 Longitude.

## Taxonomic coverage

### Description

A total of 10 families, 28 species and 9,335 individuals were collected from 71 stations during the summer and winter surveys (Table 1). St. 17 and St. 52 had the highest number of species (12) and St. 54 had the highest number of individuals (679). In contrast, fish were not detected at St. 12, St.19 and St.67. The highest number of individuals found was 2,708 in *Zaccoplatypus*, which appeared at 45 stations.

The number of individuals confirmed was 5,458 (25 species) in summer and 3,877 (21 species) in winter, with more individuals being collected in winter. In particular, migratory fish, such as *Tridentigerobscurus*, were collected in large numbers during the summer. On the other hand, 119 individuals of *Pseudogobiopolystictus* species were collected in winter compared to five individuals in summer and more were confirmed in winter. *Pseudobagrustokiensis*, *Rhinogobiusgiurinus* and *Pseudorasboraparva* were not detected in summer, whereas *Plecoglossusaltivelis*, *Poeciliareticulata*, *Cobitisbiwae*, *Micropterussalmoides*, *Lepomismacrochirus*, *Mugilcephalus* and *Pungtungiaherzi* were not collected during winter.

**Figure 2.**
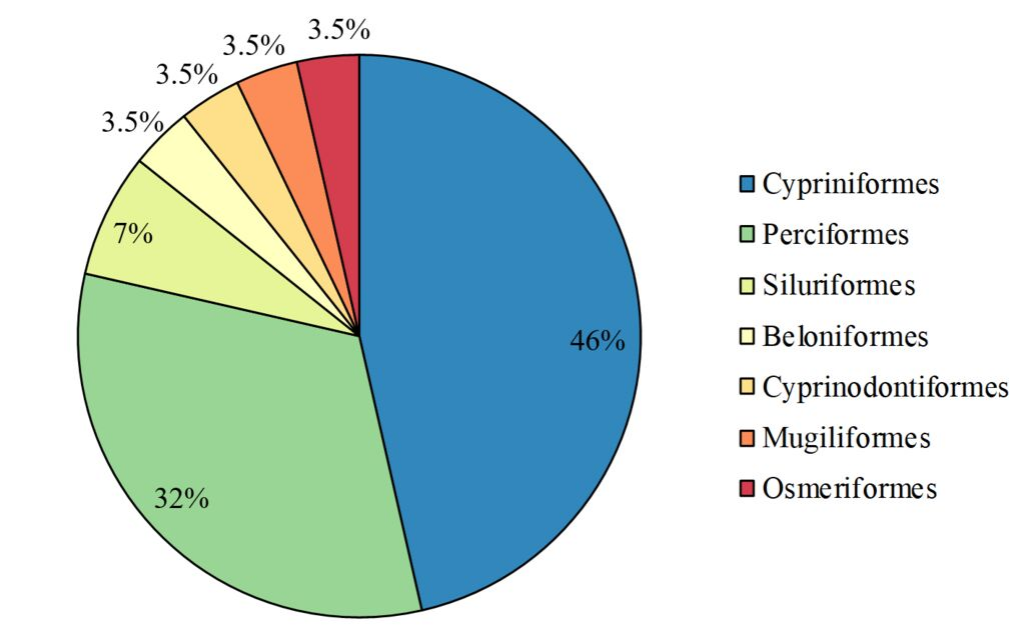
Taxonomic coverage (by order).

**Figure 3.**
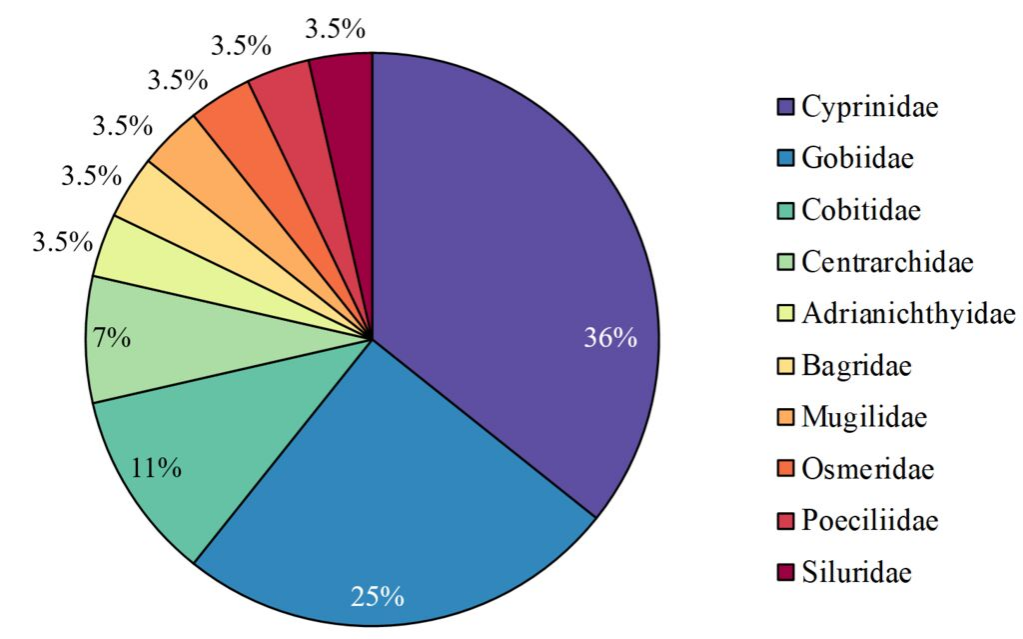
Taxonomic coverage (by family).

The species that were found belonged to the following orders: Cypriniformes (13 species), Perciformes (9 species), Siluriformes (2 species), Beloniformes (1 species), Cyprinodontiformes (1 species), Mugiliformes (1 species) and Osmeriformes (1 species) (Fig. 2). The families were Cyprinidae (10), Gobiidae (7), Cobitidae (3), Centrarchidae (2), Adrianichthyidae (1), Bagridae (1), Mugilidae (1), Osmeridae (1), Poeciliidae (1) and Siluridae (1) (Fig. 3).

According to the Red Data Book published by Kanagawga Prefecture (2006), *Tanakialanceolata* (Temminck & Schlegel, 1846) was determined to be extinct. *Oryziaslatipes* (Temminck & Schlegel, 1846) and *Pseudobagrustokiensis* (Döderlein, 1887) were determined to be critically endangered. *Lefuaechigonia* (Jordan & Richardson, 1907) was determined to be endangered. *Rhynchocyprislagowskiisteindachneri* (Dybowski, 1869), *Pseudogobiopolystictus* (Tominaga & Kawase, 2019), *Rhinogobiusgiurinus* (Rutter, 1897), *Cobitisbiwae* (Jordan & Snyder, 1901) and *Gymnogobiuspetschiliensis* (Rendahl, 1924) were determined to be Near Threatened (NT). *Silurusasotus* (Linnaeus 1758) was identified as N (noteworthy). In addition, *Cyprinuscarpio* (Linnaeus, 1758) is listed as DD (data deficient). Amongst these species, *Pseudobagrustokiensis*, *Oryziaslatipes*, *Lefuaechigonia* and *Tanakialanceolata* were also listed in the Red Data Book of the Ministry of the Environment for the entire nation (Ministry of the Environment 2020).

## Usage licence

### Usage licence

Creative Commons Public Domain Waiver (CC-Zero)

## Data resources

### Data package title

database_fish_urban_tsurumi

### Resource link

https://ipt.pensoft.net/manage/resource?r=database_fish_urban_tsurumi

### Alternative identifiers

https://www.gbif.org/dataset/dc878d3c-ebc2-4b95-824b-2209fb3f38f4

### Number of data sets

1

### Data set 1.

#### Data set name

database_fish_urban_tsurumi

#### Description

Surveys were conducted at 71 sites in the Tsurumi River Basin during summers and winters, focusing on river channels that have been straightened or converted to concrete channels due to urbanisation. As a result of this investigation, 10 families, 28 species and 9,335 individuals were collected (Itsukushma and Maruoka 2022).

**Data set 1.** 

Column label	Column description
occurrenceID	An identifier for the Occurrence.
basisOfRecord	The specific nature of the data record.
samplingProtocol	The names of, references to, or descriptions of the methods or protocols used during an Event.
eventDate	The date-time or interval during which an Event occurred.
scientificName	The full scientific name.
scientificNameAuthorship	The authorship information for the scientificName formatted according to the conventions of the applicable nomenclaturalCode.
kingdom	The full scientific name of the kingdom in which the taxon is classified.
phylum	The full scientific name of the phylum or division in which the taxon is classified.
class	The full scientific name of the class in which the taxon is classified.
order	The full scientific name of the order in which the taxon is classified.
family	The full scientific name of the family in which the taxon is classified.
taxonRank	The taxonomic rank of the most specific name in the scientificName as it appears in the original record.
identificationRemarks	Comments or notes about the Identification.
identifiedBy	A list (concatenated and separated) of names of people, groups or organisations who assigned the Taxon to the subject.
recordedBy	A list (concatenated and separated) of the globally unique identifier for the person, people, groups, or organisations responsible for recording the original Occurrence.
decimalLatitude	The geographic latitude (in decimal degrees, using the spatial reference system given in geodeticDatum) of the geographic centre of a Location.
decimalLongitude	The geographic longitude (in decimal degrees, using the spatial reference system given in geodeticDatum) of the geographic centre of a Location.
coordinateUncertaintyInMetres	The horizontal distance (in metres) from the given decimalLatitude and decimalLongitude describing the smallest circle containing the whole of the Location.
geodeticDatum	The ellipsoid, geodetic datum or spatial reference system (SRS) upon which the geographic coordinates given in decimalLatitude and decimalLongitude are based.
countryCode	The standard code for the country in which the Location occurs. Recommended best practice is to use ISO 3166-1-alpha-2 country codes.
individualCount	The number of individuals represented present at the time of the Occurrence.
occurrenceStatus	A statement about the presence or absence of a Taxon at a Location.
catalogNumber	A list (concatenated and separated) of previous or alternative fully qualified catalogue numbers or other human-used identifiers for the same Occurrence, whether in the current or any other dataset or collection.
language	A language of the resource. Recommended best practice is to use a controlled vocabulary, such as RFC 4646 [RFC4646].
country	The name of the country or major administrative unit in which the Location occurs. Recommended best practice is to use a controlled vocabulary, such as the Getty Thesaurus of Geographic Names.
stateProvince	The name of the next smallest administrative region than country (state, province, canton, department, region etc.) in which the Location occurs.
municipality	The full, unabbreviated name of the next smallest administrative region than county (city, municipality etc.) in which the Location occurs. Do not use this term for a nearby named place that does not contain the actual location.
locality	The specific description of the place. Less specific geographic information can be provided in other geographic terms (higherGeography, continent, country, stateProvince, county, municipality, waterBody, island, islandGroup). This term may contain information modified from the original to correct perceived errors or standardise the description.
modified	The most recent date-time on which the resource was changed. For Darwin Core, recommended best practice is to use an encoding scheme, such as ISO 8601:2004(E).
year	The four-digit year in which the Event occurred, according to the Common Era Calendar.
month	The ordinal month in which the Event occurred.
day	The integer day of the month on which the Event occurred.
locationID	An identifier for the set of location information (data associated with dcterms:Location). May be a global unique identifier or an identifier specific to the dataset.
informationWithheld	Additional information that exists, but that has not been shared in the given record.
dataGeneralisations	Actions taken to make the shared data less specific or complete than in its original form. Suggests that alternative data of higher quality may be available on request.

## Additional information

As a result of this investigation, 24 native species and four alien fish species were identified from the target sites. The average number of native fish species in the Kanto Plain ecological region, to which the Tsurumi River belongs, is 32.1 (Itsukushima 2019) and the survey results were lower than the average number of fish species belonging to the ecological region. Therefore, we investigated rivers that are significantly urbanised and have highly degraded habitats and the investigated rivers are relatively small to medium-sized rivers.

On the other hand, our results revealed that rare species are relatively abundant even in urban rivers that have been significantly urbanised. In particular, *Tanakialanceolata* is thought to be extinct in the wild (Kanagawga Prefecture 2006) because the species has not been confirmed in recent surveys and bivalves, which serve as spawning substrates, are not collected at all (Okitsu and Suguro 2001). However, in this survey, six individuals were collected, even from urban rivers where channel modification has progressed. Furthermore, 93 L. echigonia individuals were collected from nine sites. The sites where *Lefuaechigonia* were collected were characterised by their proximity to the few remaining forest areas in the Basin. These sites are also characterised by a concrete-covered channel; however, in the present study, species were often collected from the bottom leaf litter in slow flow areas. Since high water temperatures adversely affect the inhabitation of *Lefuaechigonia* and the importance of riverbed environments, such as sandy mud and litter, has been pointed out (Ina and Kuramoto 2003, Mitsuo et al. 2007), it is likely that a large number of individuals of species were collected near forests, which can block sunlight and add fallen leaves and fine particles to the site. In addition, the presence of *Cyprinuscarpio*, which is listed as Data Deficient in the Red Data Book of Kanagawa Prefecture, was confirmed at 25 sites and a total of 502 individuals were identified in the present study. However, considering the fact that continental introduced strains of *Cyprinuscarpio* are widespread throughout the country (Mabuchi et al. 2008) and carp releases have been implemented in the targeted watershed of the present study, it is considered that the strains are not native to Japan.

The results of this survey showed that *Zaccoplatypus* was the most frequently collected species in both summer and winter, accounting for approximately 30% of all collected individuals and was the dominant species at many sites. *Zaccoplatypus* increases in river channels that have been straightened by channel modification and have many shallow run habitats (Higuchi et al. 1997) and this fish species has been shown to be resistant to anthropogenic impacts (Sago and Nagai 2003). The dominance of this species in this study, where many of the investigation sites were environmentally degraded, supports the results of previous studies.

In general, urbanisation causes a decrease in species diversity because of the expansion of distribution areas of alien species and the subsequent loss of native species (Marchetti et al. 2006, Olden et al. 2006). However, in the present study, except for *Misgurnus* sp. (clade B2), a total of only three individuals of non-native species were collected during summer and winter, resulting in a small number of non-native fish, although the sites were significantly affected by human activities. This may be due to the fact that the depth of the water was shallow for non-native fish species to inhabit at the sites where the environment has been substantially degraded due to channelling with concrete. Moreover, there are very few water intake weirs for agricultural use near the target stations owing to the urbanisation of the entire watershed and, therefore, a waterlogged environment, which is the main habitat of carnivorous non-native fish, such as *Micropterussalmoides* and *Lepomismacrochirus*, is absent in the region.

As a result of this survey, it was evident that a relatively large number of fish species and several rare species inhabit rivers in urban areas where the environment has been substantially degraded owing to land use changes and river modifications, such as river channelling. In addition, the spread of commonly-known alien fish has not been confirmed in urbanised rivers, with the exception of a few species. Our results suggest the importance of continuing to collect in-depth data on urban rivers with degraded environments and elucidating the actual status of fish fauna in urban rivers.

## Figures and Tables

**Table 1.** Occurrence of fish taxa in the Tsurumi River Basin represented in the dataset.

Taxa	Number of occurences	native/alien	Red List Rank (Kanagawa Prefecture)
Cyprinidae			
*Cyprinuscarpio*	502	native	Data deficient (DD)
*Carassiusauratuslangsdorfii*	35	native	
*Critically Endangered*	1,043	native	Near threatened (NT)
*Zaccoplatypus*	2,708	native	
*Pseudogobiopolystictus*	124	native	Near threatened (NT)
*Nipponocypristemminckii*	205	native	
*Gnathopogonelongatuselongatus*	683	native	
*Pungtungiaherzi*	2	native	
*Pseudorasboraparva*	32	native	
*Tanakialanceolata*	6	native	Extinct (EX)
Gobiidae			
*Rhinogobiusgiurinus*	1	native	Near threatened (NT)
*Gymnogobiusurotaenia*	20	native	
*Rhinogobiusnagoyae*	45	native	
*Gymnogobiuspetschiliensis*	317	native	Near threatened (NT)
*Tridentigerobscurus*	1,114	native	
*Rhinogobius* sp.	980	native	
*Tridentigerbrevispinis*	122	native	
Cobitidae			
*Misgurnus* sp. (clade B2)	1,109	alien	
*Cobitisbiwae*	2	native	Near threatened (NT)
*Lefuaechigonia*	93	native	Endangered (EN)
Centrarchidae			
*Micropterussalmoides*	1	alien	
*Lepomismacrochirus*	1	alien	
Osmeridae			
*Plecoglossusaltivelis*	4	native	
Bagridae			
*Pseudobagrustokiensis*	1	native	Critically endangered (CR)
Poeciliidae			
*Poeciliareticulata*	1	alien	
Siluridae			
*Silurusasotus*	18	native	Noteworthy (N)
Mugilidae			
*Mugilcephalus*	3	native	
Adrianichthyidae			
*Oryziaslatipes*	163	native	Critically endangered (CR)
